# Modulating Galectin-1 in human osteoblast-like cells alters mineralisation and influences the expression of genes associated with osteoblasts and osteocytes

**DOI:** 10.1042/BSR20253890

**Published:** 2026-02-23

**Authors:** Amy C. Ross, Lauren Feddersen, Jayesh Dudhia, Androniki Psifidi, Deborah J. Guest

**Affiliations:** Centre for Vaccinology and Regenerative Medicine, Department of Clinical Sciences and Services, The Royal Veterinary College, Hawkshead Lane, North Mymms, Hatfield, Herts AL9 7TA, U.K.

**Keywords:** bone, Galectin 1, gene knockdown, gene overexpression, osteoblasts, osteocytes

## Abstract

Bone formation, mineralisation, and remodelling are dependent on the activity of osteoblasts and osteoclasts. While many genes support bone development, their specific functions in the different stages of cell differentiation and maturation are often unknown. Galectin-1 (LGALS1) has roles in tumour angiogenesis and immune regulation but also enhances osteoblast differentiation of MSCs. The present study aimed to elucidate the role of LGALS1 in the osteogenesis of Saos2 cells. We demonstrated that Saos2 cells have a gene expression profile representing a mature osteoblast stage that shifts to an osteocyte-like profile under osteogenic conditions. We modulated LGALS1 using stable transfection to overexpress LGALS1 and lentiviral transduction of a short-hairpin RNA to knock down LGALS1. LGALS1 overexpression significantly reduced cell viability under basal culture conditions and significantly reduced mineralisation after 21 days of osteogenic culture. Knockdown of LGALS1 also significantly reduced cell viability under basal culture, but following osteogenic culture, a significant increase in mineralisation was observed. No effect on collagen deposition was seen following either knockdown or overexpression, suggesting that LGALS1 is involved in negatively regulating mineralisation. Osteogenic gene expression demonstrated few significant changes when LGALS1 was overexpressed. In contrast, knocking down LGALS1 resulted in many significant changes in osteogenic gene expression under both basal and osteogenic culture conditions. However, there was no clear correlation between the effect on gene expression and the increase in mineralisation. The present study demonstrated a novel role of LGALS1 in mature osteoblast-like cells. Further investigation into the regulatory pathways is warranted.

## Introduction

Bone is a mineralised connective tissue that is one of the main components of the skeletal system and is imperative for protection of internal organs and locomotion [1]. The main cellular constituents of bone are osteoblasts, bone lining cells, osteocytes, and osteoclasts [2].

Bones undergo constant remodeling throughout adult life in response to loading and to replace damaged or old bone. This process occurs in four parts. The first phase is activation, during which M-CSF (macrophage colony stimulating factor) and RANKL (receptor activator of nuclear factor kappa-B ligand) are secreted by bone lining cells, promoting the differentiation of pre-osteoclast cells into osteoclasts [3]. Next comes the resorption phase, where mature osteoclasts resorb bone by secreting cathepsin K and H+ into a cell-matrix adhesion zone known as the sealing zone. Osteoclasts then detach and undergo apoptosis. The reversal stage follows, in which pre-osteoblasts differentiate into osteoblasts and are recruited into the resorption area. Finally, bone formation and mineralisation occur as these osteoblasts begin depositing the bone matrix, which then mineralises. After this, the bone returns to a resting state [4].

Alterations in osteoblast and osteoclast activity can therefore lead to numerous bone diseases [5]. For example, in osteoporosis resorption by osteoclasts outpaces bone formation by osteoblasts, which leads to decreased bone density and increased fracture risk [6]. In the group of genetic disorders leading to osteopetrosis, osteoclasts fail to resorb bone, which leads to dense but brittle, fragile bones that are also fracture-prone [7]. Paget’s disease of bone is characterized by excessive osteoclast activity and abnormal osteoblast activity, which produces disorganized trabecular bone [8]. Osteomalacia (rickets) due to deficiency in vitamin D or calcium, impairs osteoblast function and normal mineralisation which leads to softer, more flexible bones [9].

Osteoblasts are derived from mesenchymal stem cells (MSCs), which originate within the bone marrow [10]. RUNX2, or Runt-related transcription factor 2, is a master transcription factor that is essential for osteoblast differentiation. It is weakly expressed in MSCs, with expression increasing with commitment to the osteoblast lineage. RUNX2 knockdown in MSCs leads to decreased osteogenic differentiation and increased chondrogenic differentiation [11], whereas its overexpression leads to increased osteogenic differentiation [12]. RUNX2 is responsible for activating other genes involved in differentiation, such as transcription factor *SP7* (osterix) [13], which directs cells toward the pre-osteoblast stage but whose expression is maintained throughout the later stages of differentiation (ranging from pre-osteoblasts to osteocytes) [13,14]. Unlike RUNX2 knockdown, SP7 knockdown in MSCs leads to a reduction in both osteogenic and chondrogenic differentiation [15], highlighting the complexity of the transcriptional control of differentiation. Pre-osteoblasts then mature into immature osteoblasts, expressing *COL1A1* (collagen type 1 alpha 1) and *BGLAP* (bone gamma carboxyglutamate protein) [13]. BGLAP is the most abundant non-collagenous bone matrix protein, and its knockdown in MSCs results in reduced levels of matrix mineralisation and a down-regulation in other osteogenic genes [16].

As cells progress into the mature osteoblast stage, mineralisation and matrix organisation are stimulated. Several key proteins are involved in this process; SPP1 (osteopontin or secreted phosphoprotein 1) forms part of the non-collagenous bone matrix but is also involved in regulating bone remodelling and mineralisation [17]. IBSP (bone sialoprotein) is a non-collagenous glycoprotein, which is expressed in mineralising osteoblasts [18]. ALPL (alkaline phosphatase) becomes highly expressed and hydrolyses phosphates, promoting hydroxyapatite deposition [19]. Down-regulation of ALPL in MSCs reduces osteogenic differentiation and increases adipogenic differentiation [20]. In contrast, ENPP1 (ectonucleotide pyrophosphatase/phosphodiesterase-1) negatively regulates mineralisation by hydrolysing extracellular nucleotide triphosphates to produce inorganic pyrophosphate (PPi). PPi acts as an inhibitor of mineralisation [21], but it can also be hydrolysed by ALPL to generate phosphate (Pi), which can promote mineralisation [22]. PHOSPHO1 (bone-specific phosphatase, phosphatase, orphan 1) is expressed in mature mineralising osteoblasts [23] and promotes mineralisation by cleaving phosphoethanolamine and phosphocholine to generate inorganic phosphate (Pi) [24]. Primary osteoblasts from PHOSPHO1 knockout mice produce lower levels of matrix mineralisation but increased levels of osteocyte marker gene expression [25]. SPARC (osteonectin) also plays a role in matrix formation by participating in collagen binding and mineralisation. As cells transition to late osteoblasts/early osteocytes, PHEX (phosphatase-regulating neutral endopeptidase), MEPE (matrix extracellular phosphoglycoprotein), and DMP1 (dentin matrix protein 1) are expressed and contribute to phosphate homeostasis and mineralisation. Additionally, SOST (sclerostin) is expressed in late osteocytes and inhibits bone formation [26]. Knockdown of SOST in MSCs enhances osteogenic and chondrogenic differentiation while reducing adipogenic differentiation [27].

However, it is important to recognise that many other genes contribute to bone development, but their specific roles in different stages and cell types often remain unclear. Galectin-1 (LGALS1) has been implicated in various biological processes, including tumour angiogenesis, and it serves as a mediator in immune modulation and the motility of bone marrow stromal cells (BMSCs) [28,29]. However, Chen and Liu [28] demonstrated that *LGALS1*^−/−^ mice at 3 months old exhibit a significant decrease in trabecular bone volume, thickness, and number of trabeculae, with trabecular space significantly increasing in the lumbar vertebrae. Cortical bone displayed a significant decrease in bone area fraction and thickness in the femur. Calcium staining revealed a significant reduction in the bone formation rate, suggesting an attenuation of new bone formation. Furthermore, Xu and Ni [30] demonstrated that a decline in LGALS1 levels in aged mice was correlated with decreases in trabecular bone thickness and number of trabeculae and increased bone space. Similarly, Chen and Liu [28] demonstrated that BMSCs from older mice showed down-regulation of *LGALS1* and a lower osteogenic differentiation capacity that could be increased by the over-expression of *LGALS1*. Furthermore, extracellular LGALS1 has been shown to inhibit osteoclast differentiation [31]. Together, these studies demonstrate a role for LGALS1 during development and stem cell differentiation; however, the functional role of LGALS1 in committed osteogenic cell types remains unknown. The present study aimed to understand the functional role of LGALS1 in osteoblasts and osteogenesis.

## Materials and methods

### Saos2 cell culture

Saos2 cells (RRID:CVCL_0548; HTB-85 from ATCC, U.S.A.) were cultured in basal growth media consisting of McCoys 5A; high glucose (3000 mg/l) without sodium pyruvate but with l-glutamine (219.2 mg/l) with the addition of 15% fetal bovine serum (FBS), 1000 U/ml penicillin, and 100 μg/ml streptomycin (P/S) (All Thermo Fisher, U.S.A.). Cells were passaged when 70%–80% confluency was reached using 0.25% trypsin–EDTA. For the analysis of cells under basal conditions, cultures were harvested at 70%–80% confluency to ensure the cells were in a proliferative state.

To differentiate the Saos2 cells, they were first cultured to 70%–80% confluency in basal media, and then the media was changed to osteogenic media and the cells cultured for up to 21 days [32] to allow cell differentiation, maturation, and bone formation. Osteogenic media was as per basal media but supplemented with 10 nM dexamethasone, 280 μM l-ascorbic acid, and 2 mM β-glycerophosphate (All Sigma, U.K.) [33,34]. The concentration of β-glycerophosphate was optimised by testing six concentrations (2, 3, 4, 5, 7, and 10 mM), and 2 mM β-glycerophosphate was selected as the lowest dose, which produced high levels of mineralisation (Supplementary Figure S1). Osteogenic media was changed every 3–4 days for the 21 days before cells were used for downstream assays.

### LGALS1 overexpression

Saos2 cells were seeded onto a 6-well plate in basal media lacking penicillin/streptomycin and cultured until 70%–80% confluent and then transfected with 2.5 μg of pCMV6-AC-GFP human *LGALS1* per well (RG204674, NM_002305; Origene, U.K.) using Lipofectamine 3000 (L3000) (Invitrogen) according to the manufacturer’s instructions. Selection was carried out 48 h post transfection with 1 mg/ml of G418 (Sigma–Aldrich, dose optimised by kill curve on non-transfected cells), and resistant cells were expanded to create an *LGALS1* overexpressing cell line.

### LGALS1 knockdown

HEK293T cells (RRID:CVCL_0063) were cultured in Dulbecco’s modified Eagle’s medium (DMEM; high glucose [4500 mg/l] with sodium pyruvate [110 mg/l]), 10% FBS, and 2 mM L-glutamine (LQ) (all Gibco, Thermo Fisher Scientific). Cells were passaged when 70%–80% confluency was reached using 0.25% trypsin–EDTA. For transfection, HEK293T packaging cells were seeded onto a 6-well plate at a density of 1 × 10^5^ per well. Twenty-four hours post-seeding, each well was transfected with a combination of 1 μg lentiviral TRC-pLK0 plasmid, 750 ng psPAX2, and 250 ng pMD2.G using FuGENE 6 transfection reagent (Promega, U.K.) according to the manufacturer’s instructions. The lentiviral plasmid used was either TRC1-pLK0.1 containing a non-target, scrambled (NT) shRNA (5′-GCGATAGCGCTAATAATTT-3′ SHC202; Sigma–Aldrich) or an shRNA specific for human *LGALS1* (L-423) (5′-GCTGCCAGATGGATACGAATT-3′ clone NM_002305; Sigma–Aldrich). A positive control, TRC2-pLKO.5-puro-CMV-TurboGFP plasmid (SHC 203; Sigma–Aldrich), was also used to visualise successful transfection and transduction. pMD2.G and psPAX2 were a gift from Didier Trono (Addgene plasmids #12259 and 12260; http://n2t.net/addgene:12259 http://n2t.net/addgene:12260; RRID: Addgene_12259 and 12260). Twenty-four hour post-transfection, media was changed to Saos2 cell basal growth media (without penicillin/streptomycin), and 48 h later packaging cell supernatant was harvested, filtered through a 0.45-μm filter (Millipore, Billerica, US), combined with 10 μg/ml polybrene (Sigma–Aldrich) and immediately applied to Saos2 cells, which were plated at 2 × 10^5^ cell density per well 24 h before transduction. One round of viral infection was carried out for 24 h, which resulted in ∼80% of the cells being transduced. Seventy-two hours after the transduction, media containing 2.5 μg/ml puromycin (Sigma–Aldrich, dose selected using a kill curve on non-transduced cells) was used to select stably transduced cells.

### LGALS1 ELISA

LGALS1 concentrations were measured in cell culture supernatant taken from three independent experimental replicates (i.e. cells at different passages set up and harvested on different dates to allow an estimate of the consistency of the effects across different experimental runs [35]) of unaltered Saos2 cells and Saos2 cells modified to both under- and over-express *LGALS1*. Supernatants were collected from cells cultured under basal conditions and after 21 days of osteogenic culture. Measurements were obtained for three experimental replicates of each condition using the human Galectin-1 Quantikine ELISA Kit (R&D Systems, U.K.) according to the manufacturer’s protocol. Each replicate was measured in duplicate using colorimetric detection at 450 nm with a background correction read at 540 nm on a Tecan plate reader (TECAN infinite M nano +, Switzerland).

### Cell viability and staining

To measure cell viability, Saos2 cells were seeded onto a 96-well tissue culture plate at a density of 1 × 10^4^ cells/well in basal media and allowed to attach at 37°C and 5% CO_2_ for 48 h. Following 48 h incubation, culture media was removed, and 100 μl of diluted PrestoBlue™ reagent (1:10; Invitrogen, Thermo Fisher) was added to each well, and the plates were incubated at 37°C for 10 min. Fluorescence for individual wells was measured using a Tecan plate reader (TECAN infinite M nano +) at 560/590 nm excitation and emission wavelengths, respectively. Viability was measured in seven technical replicates (independent wells) and is shown in relative fluorescent units (RFU) as a measure of the amount of resorufin (produced from the reduction in resazurin by viable cells).

To assess morphology, three experimental replicates of Saos2 cells were cultured on gelatin (Sigma)-coated coverslips in 24-well plates. Cells were fixed using 3% paraformaldehyde (Sigma) for 20 min at room temperature prior to washing in phosphate buffered saline (PBS, Sigma). Fixed cells were permeabilised with 0.1% Triton-X-100 (Sigma) for 1 h at room temperature, followed by three washes in PBS and blocking with 2.5% normal horse serum (NHS, 2BScientific, Oxfordshire, U.K.) for 20 min at room temperature. Coverslips were incubated with 50 μl phalloidin conjugated to Alexa Fluor 594 (1:500; ab176757, Abcam, Cambridge, U.K.) diluted in 2.5% NHS for 3 h at room temperature. Vectashield HardSet was used to mount the coverslips onto glass slides. Images were captured using a Nikon Eclipse Ti2 series microscope (Nikon, Surrey, U.K.). Cell area and circularity were measured using ImageJ (National Institutes of Health, U.S.A.; ImageJ (RRID:SCR_003070)). Circularity was calculated using (4∙π (A/P^2^)), where P is the perimeter and A is the cell area. A circularity value of 1 indicates a perfect circle, while a value of 0 indicates an elliptical shape. A minimum of 20 individual cells were measured.

### RNA extraction, cDNA synthesis, and qPCR

RNA was harvested using 1 ml of Tri Reagent (Sigma) per 10 cm plate of Soas2 cells. To extract RNA, the Qiagen RNeasy kit was used (Qiagen, Hilden, Germany), and any genomic DNA contaminants were removed using the Invitrogen™ DNA-free™ DNA Removal Kit (Thermo Fisher) according to the manufacturer’s instructions. Isolated RNA concentrations were calculated using a NanoDrop™ 1000 spectrophotometer (Thermo Fisher). 260:280 ratios were an indicator of the purity level of the sample and confirmed to be approximately 2.0. To produce cDNA, 1 μg of RNA was reverse transcribed using the SensiFAST cDNA Synthesis Kit (Bioline, London, U.K.) on a Biorad T100 Thermal Cycler (Bio-Rad, Hertfordshire, U.K.) according to the manufacturer’s instructions. Two microlitres of cDNA (20 ng) were used per reaction with Sensimix SYBR Green supermix (Bioline) on a Biorad C1000 Touch Thermal Cycler (Bio-Rad). Reactions were performed in duplicate. qPCR cycle constraints were 95°C for 10 min followed by 45 cycles of 95°C (15 s), 60°C (15 s), and 72°C (15 s). Following this, a melt curve was generated with readings taken every 1°C from 65°C to 95°C. A housekeeping gene was used to quantify the relative gene expression of candidate genes. The calculation 2^−ΔCT^ [36] was used to determine relative gene expression. Four housekeeping genes (glyceraldehyde 3-phosphate dehydrogenase (*GAPDH*), β-12 actin (*ACTB*), beta-2-microglobulin (*B2M*) and ribosomal protein S18 (*RPS18*)) were tested for their stability across the experiments using RefFinder [37] (https://www.heartcure.com.au/reffinder), and *ACTB* was selected for use (results in Supplementary Table S1). Primers to human genes were designed using NCBI Primer Blast (https://www.ncbi.nlm.nih.gov/tools/primer-blast/) and mfold (http://www.unafold.org/) to produce amplicons of 50–150 bp, a melting temperature of 58°C–62°C and devoid of secondary structures at Tm 60°C. The primer sequences are provided in Supplementary Table S2. Relative gene expression following LGALS1 modulation was summarised in heatmaps created in GraphPad Prism version 10.1.2(324) using *Z*-score normalisation across either all basal gene expression data or all osteogenic gene expression data. Gene expression was measured in three to six independent, experimental replicates.

### Alkaline phosphatase activity

Alkaline phosphatase activity in cell culture supernatant was measured in three independent experimental replicates using a quantitative fluorometric assay (Abcam, Cambridge, U.K.) according to the manufacturer’s instructions. Fluorescence was measured on a TECAN infinite M nano + (Tecan) plate reader Ex/Em = 360/440 nm. Culture supernatants were collected from Saos2 cells grown under basal conditions and after 7, 14, and 21 days of osteogenic culture. All culture media was collected 48–72 h post-media change.

### Mineralisation assays

Saos2 cells were cultured in 24-well plates under basal conditions until they reached 70%–80% confluency or under osteogenic culture conditions for 21 days. They were fixed using 3% paraformaldehyde for 20 min at room temperature prior to washing three times in PBS and staining. For the detection of calcium deposition, fixed cells were stained with 2% Alizarin red S pH 4.2 (Sigma) for 5 min at room temperature. Excess stain was washed off with water, and the cells were imaged using an EVOS XL core microscope (Thermo Fisher) with a 4× objective.

For the detection of mineralised calcium phosphate crystals, fixed cells were stained using the OsteoImage kit (Lonza, Slough, U.K.), following the manufacturer’s instructions. Fluorescent staining was visualised using a fluorescent microscope (EVOS FL, Thermo Fisher) on the GFP channel, and images were captured with a 4× objective.

For the detection of collagen, fixed cells were stained using a Picrosirius Red stain kit (Abcam, ab150681). Briefly, the fixed cells were hydrated with distilled water and then stained with picrosirius red solution for one hour at room temperature. After incubation, cells were rinsed with acetic acid solution and absolute alcohol, followed by a Histoclear (Scientific Laboratory Supplies, Nottingham, U.K.) rinse and then mounted using Vectamount permanent mounting medium (Vector Laboratories). Staining was visualised on a Nikon Eclipse Ti2 series microscope (Nikon, Surrey, U.K.), and images were captured with a 4× objective.

For all staining, a minimum of three images were taken across a minimum of three independent, experimental replicates, and all images were analysed using ImageJ (National Institutes of Health, U.S.A.) to quantify the percentage coverage of positive staining.

### Statistical analysis

Statistical analysis was executed using GraphPad Prism (version 10.1.2(324)). All data sets were examined for Gaussian distribution using the Shapiro–Wilks normality test; equal variance was examined using an F test. If data had equal variance, an unpaired *t*-test was performed; if equal variance could not be assumed, an unpaired *t*-test with Welsh’s correction was performed. If data were not normally distributed, data were log transformed, but if normality was still not met, a Mann–Whitney U test was performed.

Data that had more than two groups was tested for normality and equal variance. If both metrics could be assumed, data were analysed using a one-way ANOVA with Tukey’s post hoc test. If equal variance could not be assumed, a Brown-Forsythe ANOVA with Games–Howell post hoc test was performed. If data were not normally distributed, a Kruskal–Wallis with Dunn’s post hoc test was used.

For data that were measured over time, a mixed effects model with Bonferroni correction was used. Significance is indicated in all instances as a *P* <0.05.

## Results

### Saos2 cells represent mature osteoblasts

**Figure 1:**
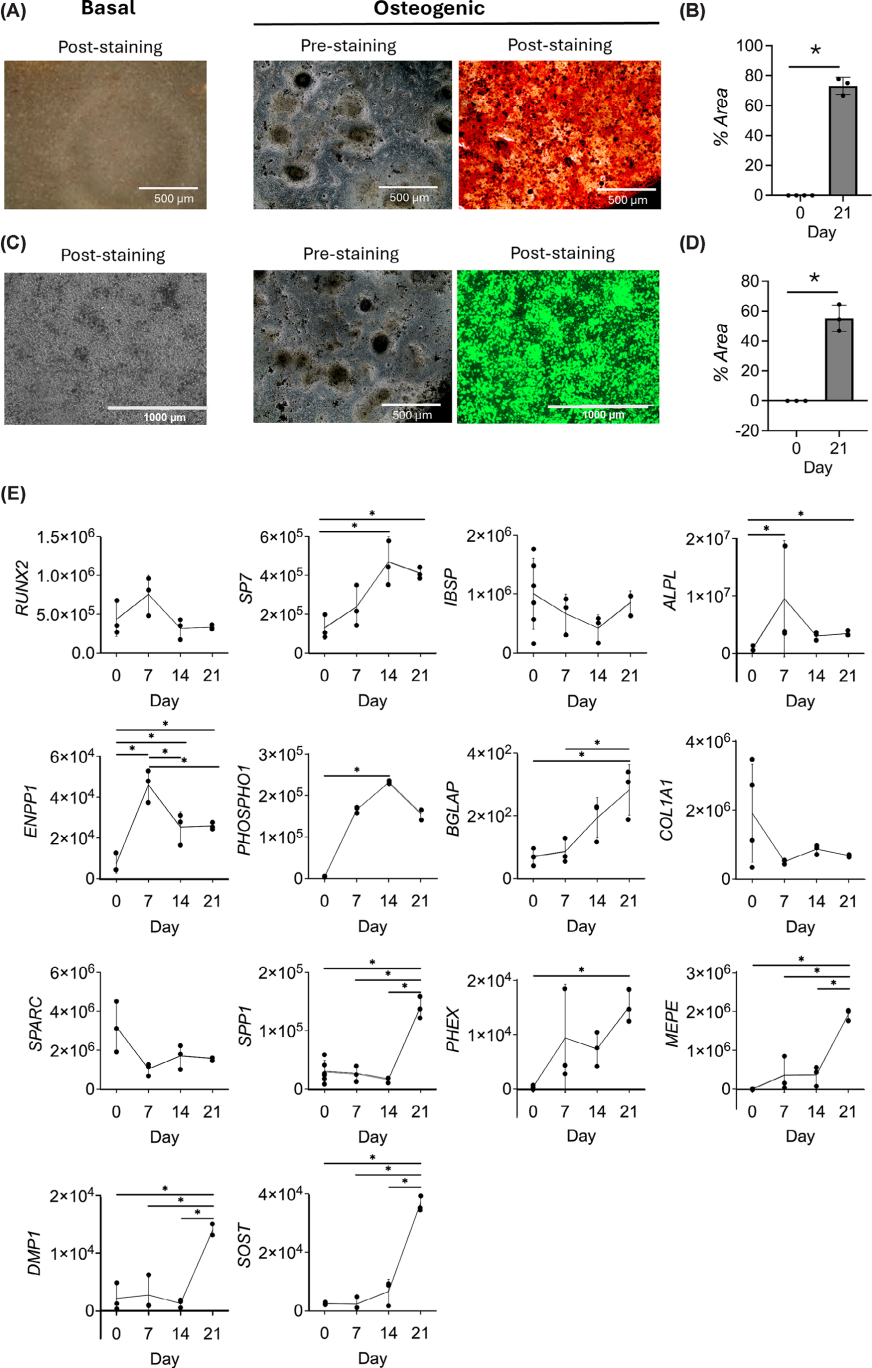
Validation of Saos2 cell line as a model for bone differentiation (**A**) Alizarin red staining of Saos2 cells in basal and after 21 days of osteogenic culture. Calcium deposits are stained red. No staining is observed under basal culture. After osteogenic culture pre- and post-staining images are shown. Scale bars = 500 μm. (**B**) Quantification of alizarin red staining as a percentage of surface area stained. Error bars represent standard deviation from three experimental replicates. * indicates *P* <0.05 (Student’s unpaired *t*-test with Welsh’s correction). (**C**) Staining of mineralised calcium phosphate crystals (green) of Saos2 cells in basal and after 21 days of osteogenic culture conditions. No staining is observed under basal culture. After osteogenic culture, pre- and post-staining images are shown. Scale bars = 500 and 1000 μm. (**D**) Quantification of staining of mineralised calcium phosphate crystals as a percentage of surface area stained. Error bars represent standard deviation from three experimental replicates. * indicates *P* <0.05. (Student’s unpaired *t*-test with Welsh’s correction). (**E**) Gene expression after 0, 7, 14, and 21 days of osteogenic culture. Expression is shown relative to the *ACTB* housekeeping gene. Error bars represent standard deviation from three to six experimental replicates. * indicates *P* <0.05 (one-way ANOVA with Tukey’s post hoc test for all genes except *IBSP* and *SPARC*, for which a Kruskall–Wallis with Dunn’s post hoc test was used).

We characterised Saos2 cells under both basal and osteogenic culture conditions. Under basal culture, the cells produce no mineralised matrix (Figure 1A,C), but under osteogenic culture, increasing amounts of mineralisation are detected over time (Supplementary Figure S2), and by 21 days, high levels of calcium and mineralised calcium phosphate crystals are observed (Figure 1A–D). Numerous changes in the expression of osteogenic genes were observed during osteogenic culture, with significant increases in the expression of *SP7, ALPL, ENPP1, PHOSPHO1, BGLAP, SPP1, PHEX*, *MEPE*, *DMP1*, and *SOST* (Figure 1E). In contrast, the expression levels of *RUNX2*, *IBSP*, *COL1A1*, and *SPARC* remained fairly consistent throughout basal and osteogenic culture. Together, the data demonstrates that when cultured under basal conditions, Saos2 cells exhibit robust expression of genes associated with a late osteoblast cell type, and when cultured under osteogenic conditions, Soas2 cells show a transition towards an osteocyte-like phenotype, with an increased expression of many late osteoblast and osteocyte-associated genes.

### LGALS1 levels can be successfully modulated in Saos2 cells through stable over-expression and shRNA mediated knock-down

**Figure 2:**
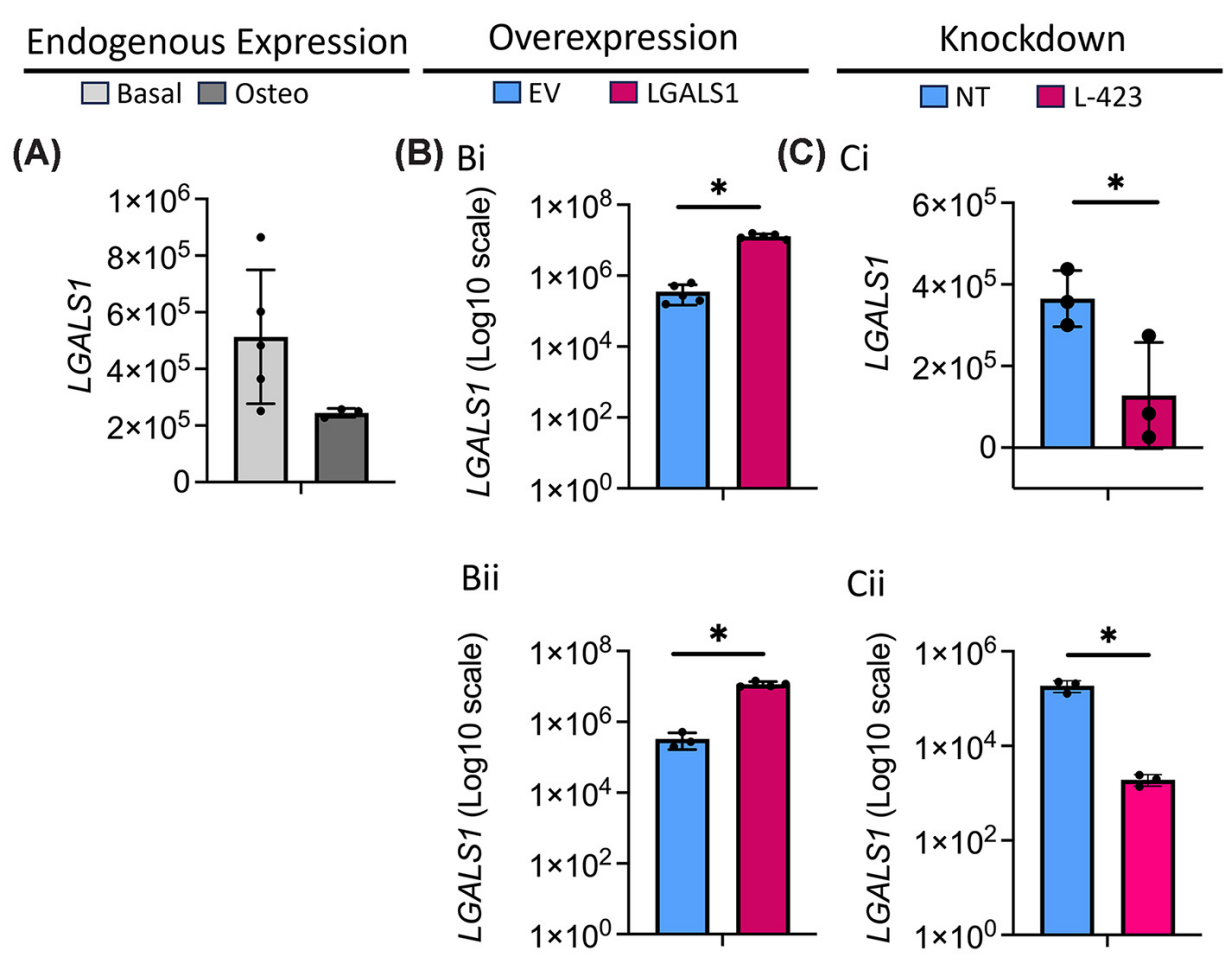
*LGALS1* gene modulation in Saos2 cells (**A**) Endogenous *LGALS1* expression in unmodified Saos2 cells cultured under basal (light grey) and osteogenic (dark grey) conditions. Overexpression (**B**) and knockdown (**C**) of *LGALS1* at the gene level under basal (i) and osteogenic (ii) culture conditions. EV = empty vector, LGALS1 = *LGALS1* overexpressing cells, NT = non-target control, L-423 = *LGALS1* knockdown. The error bars represent the standard deviation from three to five experimental replicates. * indicates *P* <0.05 (Student’s unpaired *t*-test, with Welsh’s correction applied to all except panel (Ci)).

Endogenous *LGALS1* is robustly expressed in Soas2 cells cultured under both basal and osteogenic conditions (Figure 2A). However, stable transfection of a plasmid encoding *LGALS1* under the control of the CMV promoter resulted in a significant increase in approximately 36-fold under basal conditions (Figure 2Bi) and 35-fold under osteogenic conditions (Figure 2Bii). This was confirmed at the secreted protein level (Supplementary Figure S3A), although variability in LGALS1 protein levels after stable over-expression led to an increase that was not statistically significant, despite all replicates showing higher levels than the control cells.

Lentiviral transduction of an shRNA targeting *LGALS1* led to a significant reduction in expression of approximately 3-fold under basal conditions (Figure 2Ci) and a 77-fold decrease under osteogenic conditions (Figure 2Cii). This was confirmed at the secreted protein level under osteogenic conditions (Supplementary Figure S3Bii). Under basal conditions, levels of secreted LGALS1 protein were very low, even in the control empty vector cells (Supplementary Figure S3Bi).

The effect of the modulations themselves was determined by measuring cell viability, gene expression, and matrix mineralisation in unaltered Saos2 cells, Saos2 cells expressing the empty vector (control for the overexpression studies), and Saos2 cells expressing the non-target (control for the knockdown studies). This demonstrated that the control vectors had no effect on cell viability (Supplementary Figure S4), but they both decreased the level of mineralisation (Supplementary Figure S5) and reduced the expression of some of the osteogenic genes under both basal culture (Supplementary Figure S6) and after 21 days of osteogenic culture (Supplementary Figure S7).

### LGALS1 modulation has significant effects on cell viability

**Figure 3:**
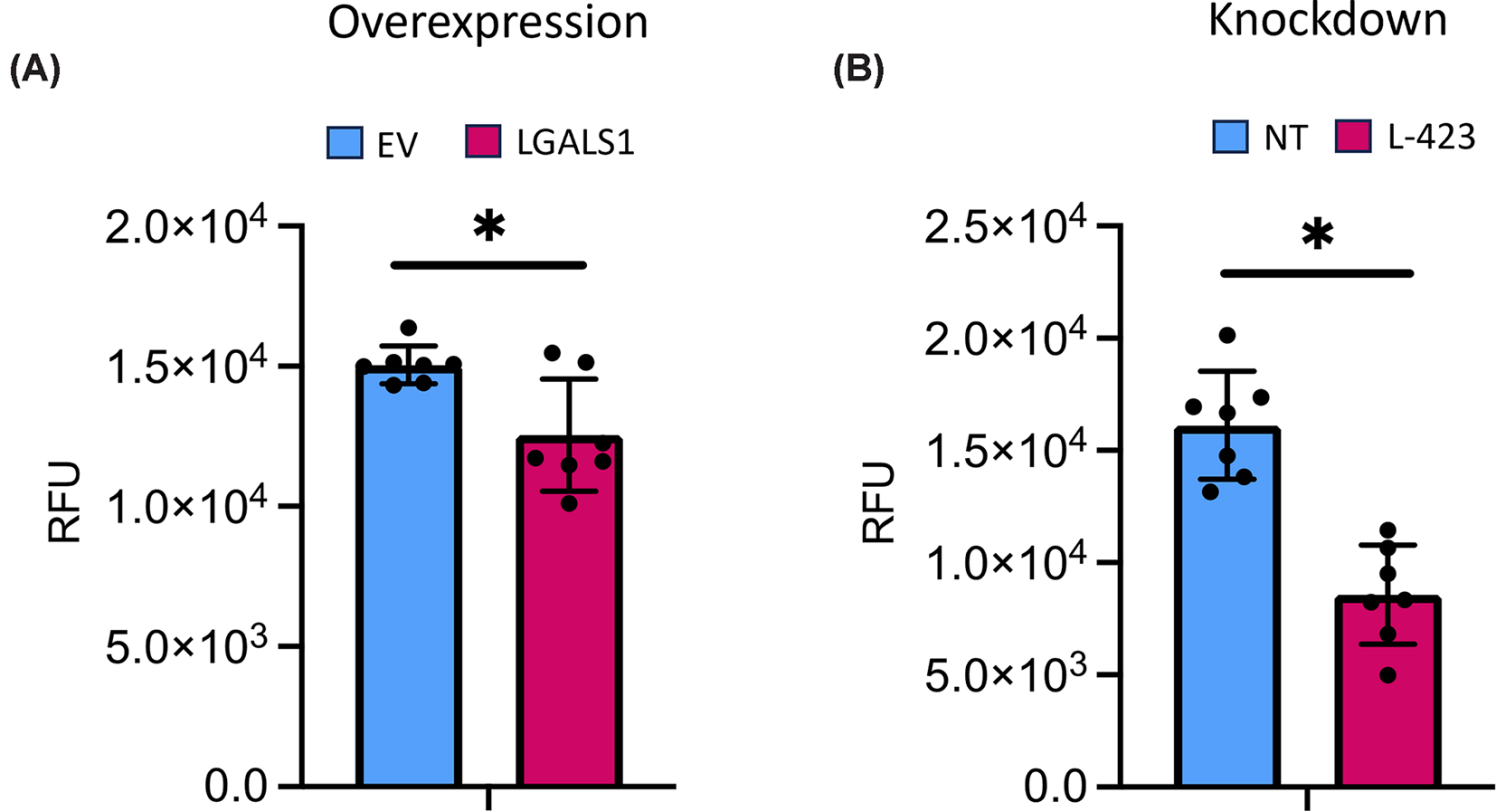
Modulation of LGALS1 affects the viability of Saos2 cells cultured under basal conditions Cell viability is shown as RFU following (**A**) *LGALS1* over-expression (EV = empty vector control, LGALS1 = *LGALS1* overexpression) and (**B**) *LGALS1* knockdown (NT = non-target control, L423 = shRNA to *LGALS1*). Error bars represent standard deviation from seven technical replicates. * indicates *P* <0.05 (Student’s unpaired *t*-test, with Welsh’s correction applied in panel (A)).

Cell viability was measured following both overexpression and knockdown of *LGALS1* under basal culture. Overexpression of *LGALS1* led to a small (17%) but significant reduction in cell viability. Knocking down *LGALS1* resulted in a significant and large (47%) reduction in viability (Figure 3).

**Figure 4:**
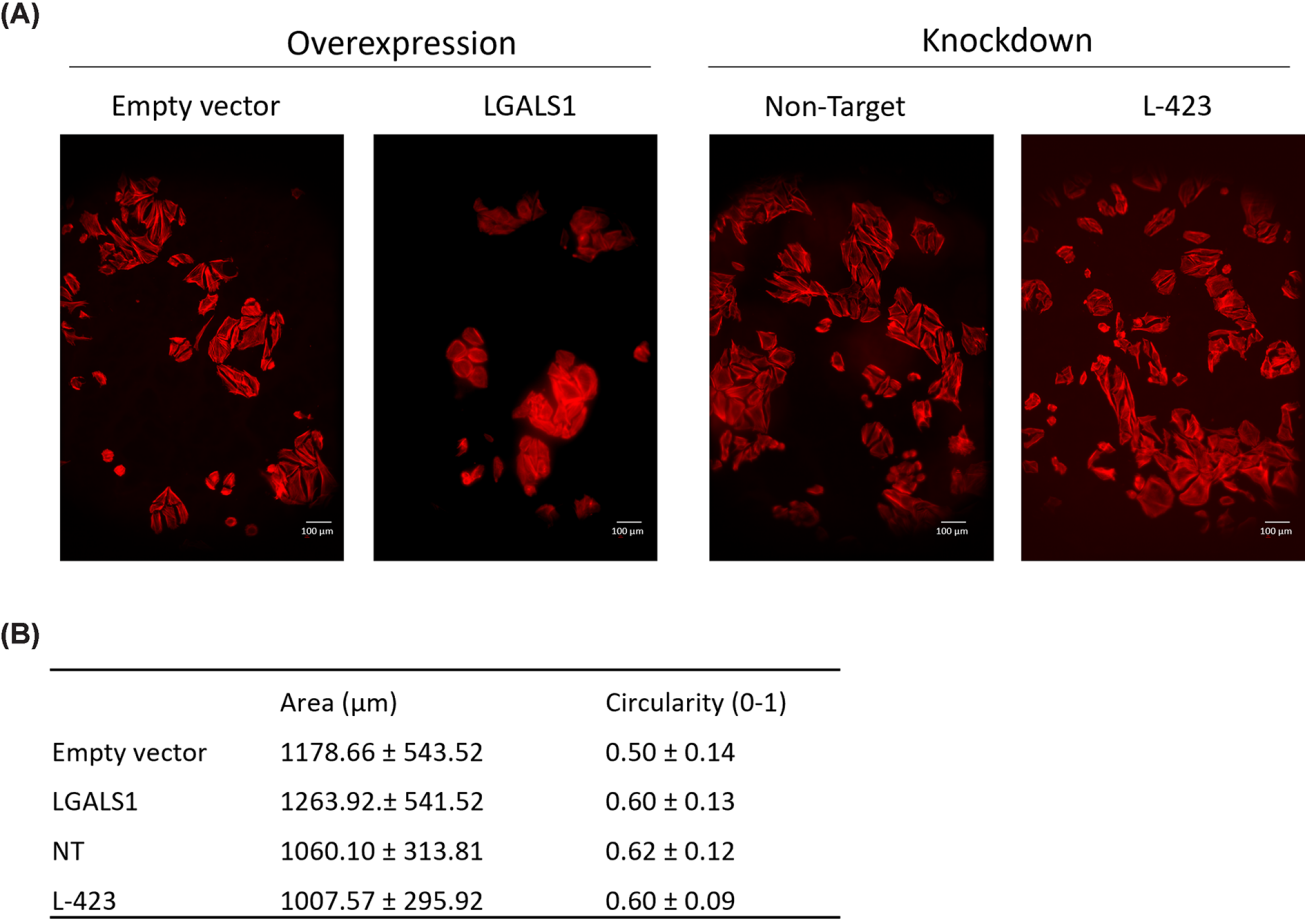
Modulation of LGALS1 has no effect on cell morphology in Saos2 cells cultured under basal conditions (**A**) Phalloidin staining (red) in empty vector control cells, LGALS1 overexpressing cells, non-target control cells, and LGALS1 knock-down cells (L-423). Scale bars = 100 μm. Images representative of three experimental replicates. (**B**) Quantification of cell area and circularity showing the mean ± standard deviation. No statistical differences were observed (one-way ANOVA).

However, there were no significant changes in cell morphology under basal conditions following either *LGALS1* overexpression or knockdown (Figure 4). It is not possible to measure cell morphology following osteogenic culture due to the confluency of the cells and the formation of the mineralised matrix.

### LGALS1 modulation in Saos2 cells significantly affects the expression of osteoblast and osteocyte-associated genes

**Figure 5:**
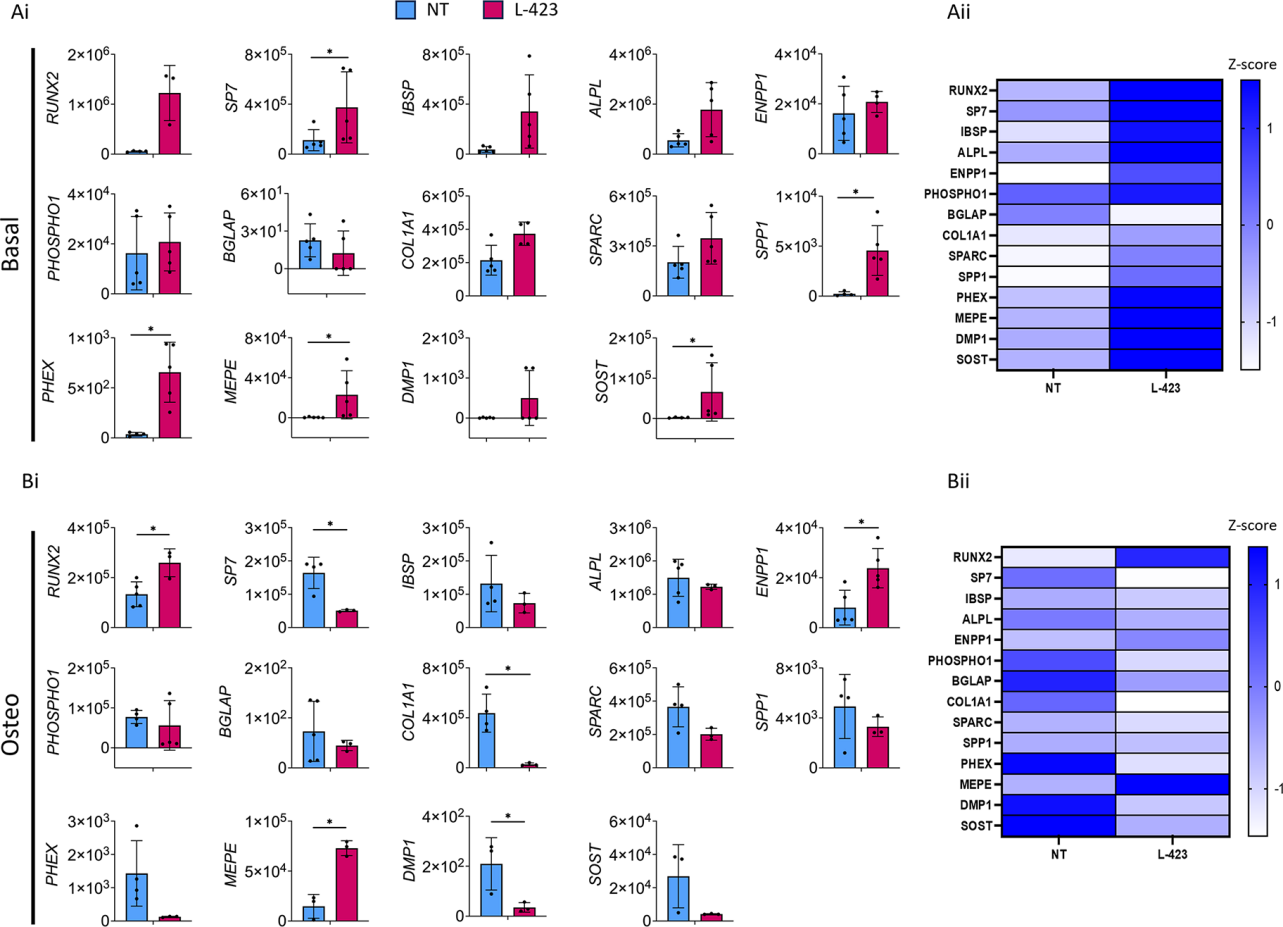
Expression of osteogenic marker genes in Saos2 cells cultured under basal and osteogenic conditions following *LGALS1* knockdown Panels (**A**i) and (**A**ii) represent basal conditions, and panels (**B**i) and (**B**ii) represent osteogenic conditions. Blue bars represent the non-target control (NT), and pink bars represent L-423 (*LGALS1* knockdown). Expression is shown relative to the *ACTB* housekeeping gene. Error bars represent the standard deviation from three to five experimental replicates. * indicates *P* <0.05 (unpaired Student’s *t*-test with or without Welsh’s correction, or where the expression was not normally distributed, a Mann–Whitney U test was performed). Heatmaps show the mean expression levels of osteogenic marker genes using *Z*-score normalisation. Blue indicates high expression, and white indicates low expression. The mean of three to five experimental replicates was used for these comparisons.

When *LGALS1* is knocked down, a trend of increased gene expression is observed under basal conditions. *SP7* (3-fold increase), *SPP1* (124-fold increase), *PHEX* (18-fold increase), *MEPE* (58-fold increase), and *SOST* (29-fold increase) all show a significant rise in expression (Figure 5Ai,ii).

Conversely, under osteogenic conditions, all genes except *RUNX2*, *ENPP1*, and *MEPE* (which exhibit significant increases of 2-fold, 3-fold, and 5-fold, respectively, in expression) display a general trend towards decreasing gene expression. For *COL1A1* (16-fold decrease), *DMP1* (6-fold decrease), and *SP7* (3-fold decrease), the reduction in expression is significant (Figure 5Bi,ii).

**Figure 6:**
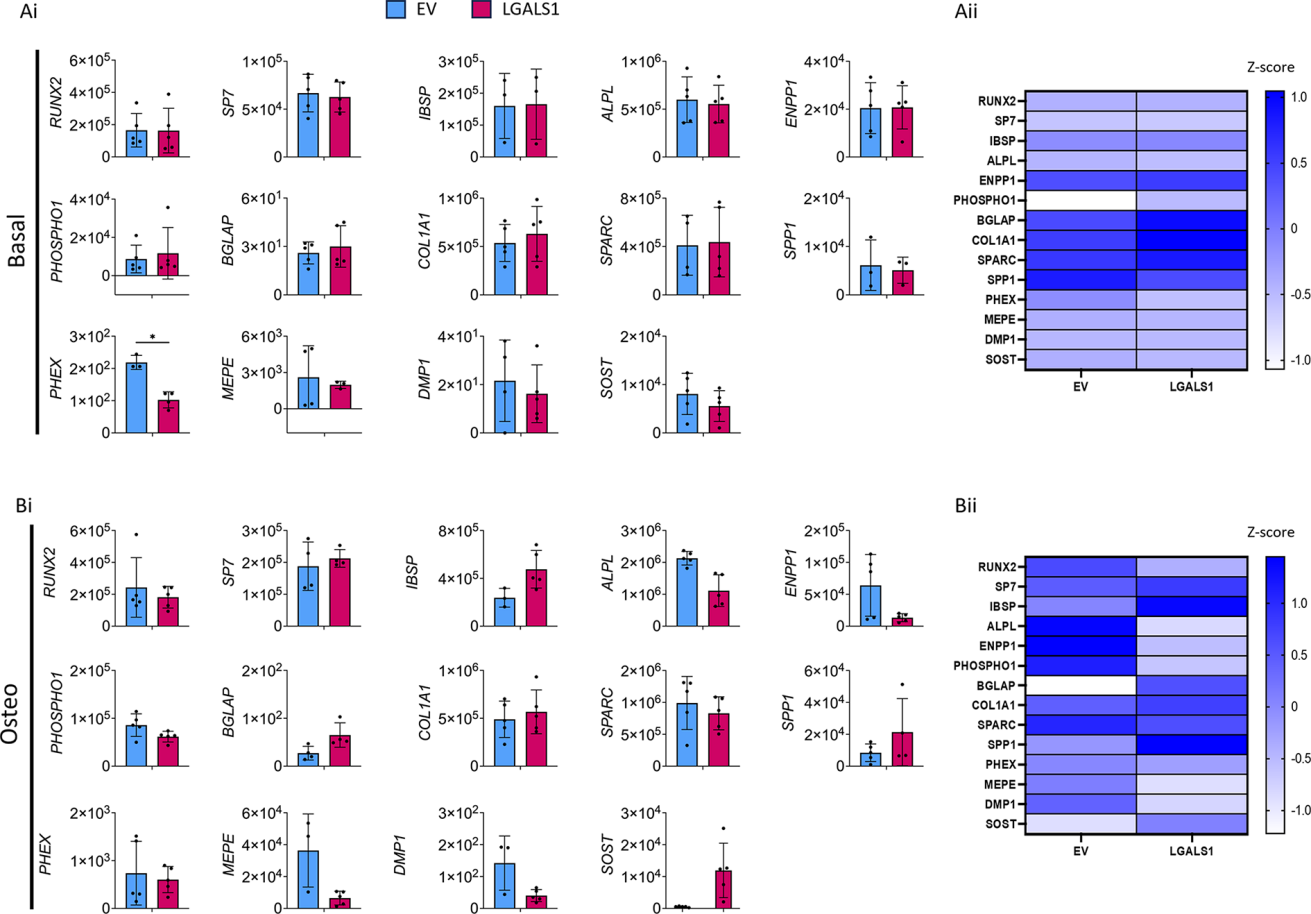
Expression of osteogenic marker genes in Saos2 cells cultured under basal and osteogenic conditions following *LGALS1* overexpression Panels (**A**i) and (**A**ii) represent basal conditions, and panels (**B**i) and (**B**ii) represent osteogenic conditions. Blue bars represent the empty vector control (EV), and pink bars represent LGALS1 (*LGALS1* overexpression). Expression is shown relative to the *ACTB* housekeeping gene. Error bars represent the standard deviation from three to five experimental replicates. * indicates *P* <0.05 (unpaired Student’s *t*-test with or without Welsh’s correction, or where the expression was not normally distributed, a Mann–Whitney U test was performed). Heatmaps show the mean expression levels of osteogenic marker genes using *Z*-score normalisation. Blue indicates high expression, and white indicates low expression. The mean of three to five experimental replicates was used for these comparisons.

In contrast, the overexpression of *LGALS1* resulted in very few changes in gene expression. Under basal culture, there was a significant two-fold decrease in the late osteoblast/osteocyte marker *PHEX*, but no other significant changes in expression were observed in either basal or osteogenic culture (Figure 6).

### LGALS1 overexpression reduces mineralisation during osteogenic culture

**Figure 7:**
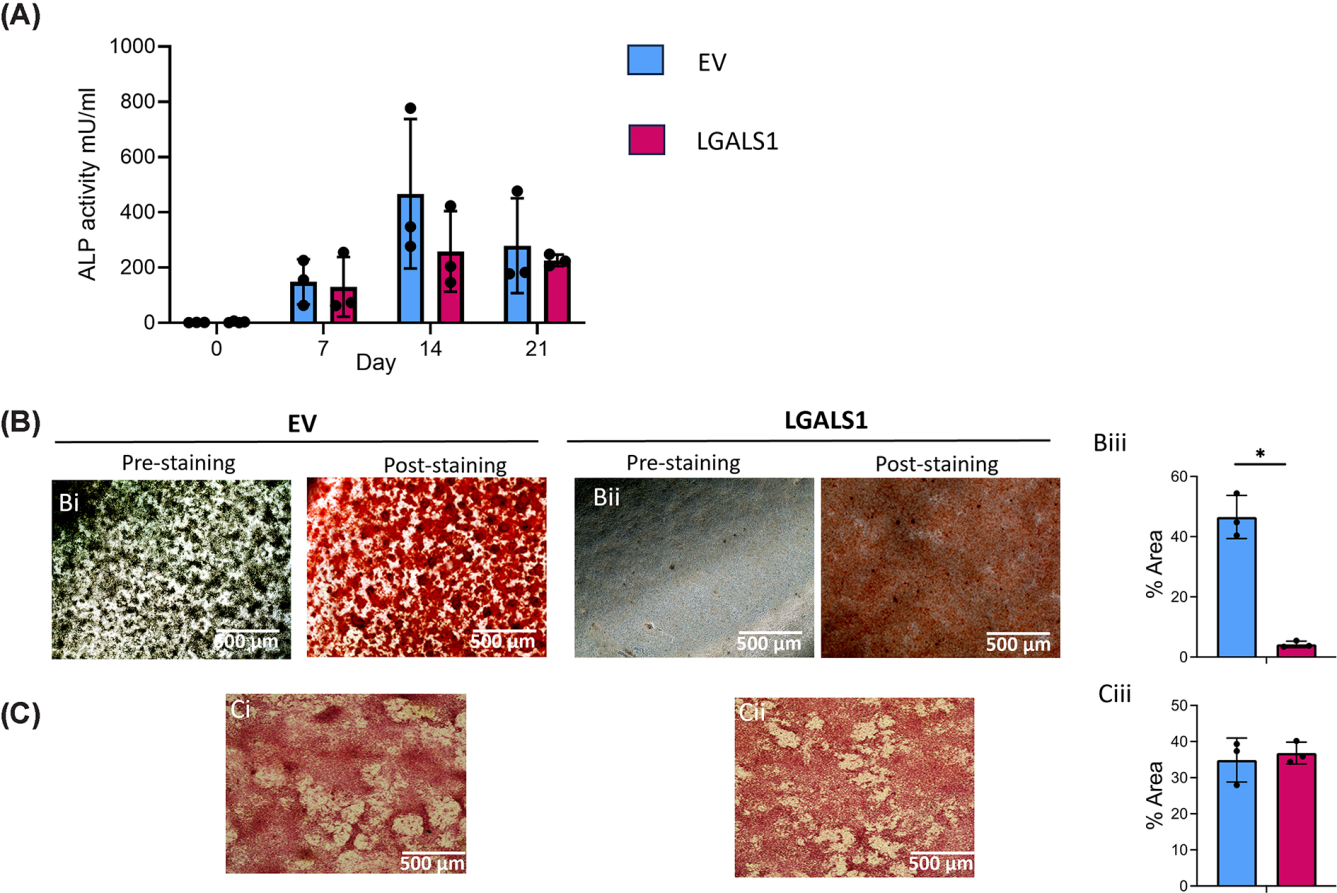
The effect of *LGASL1* overexpression on ALP activity, mineralisation, and matrix deposition (**A**) ALP activity measured in conditioned media taken at day 0 (basal culture) and after 7, 14, and 21 days in osteogenic media (EV = empty vector control, LGALS1 = *LGALS1* overexpressing cells). No significant differences were observed (mixed effects model with Bonferroni correction). (**B**) Alizarin red staining for calcium deposits after 21 days of osteogenic culture shown pre- and post-staining in (i) empty vector control cells and (ii) cells overexpressing *LGALS1*. (iii) Quantification of alizarin red staining showing the percentage area that is positively stained. * indicates *P* <0.05 (Student’s unpaired *t*-test with Welsh’s correction) (**C**) Picrosirius red staining for collagen deposition after 21 days of osteogenic culture in (i) empty vector control cells and (ii) cells overexpressing *LGALS1*. (iii) Quantification of Picrosirius red staining showing the percentage area that is positively stained. Error bars represent standard deviation from three experimental replicates. No significant differences were observed (Student’s unpaired *t*-test). Scale bars = 500 μm.

Overexpression of *LGALS1* does not lead to any significant changes in ALP activity during basal or osteogenic culture at any time point examined (Figure 7A). However, the level of matrix deposition (as measured using alizarin red) after 21 days of osteogenic culture demonstrates a significant reduction of 11-fold in cells overexpressing *LGALS1* (Figure 7Bi–iii). Since these cells express GFP, it was not feasible to conduct OsteoImage staining of mineralised calcium phosphate crystals, which generates a green fluorescent signal. Picrosirius red staining for collagen matrix deposition reveals no significant changes after *LGALS1* overexpression (Figure 7Ci–iii).

### LGALS1 knockdown increases mineralisation deposition during osteogenic culture

**Figure 8:**
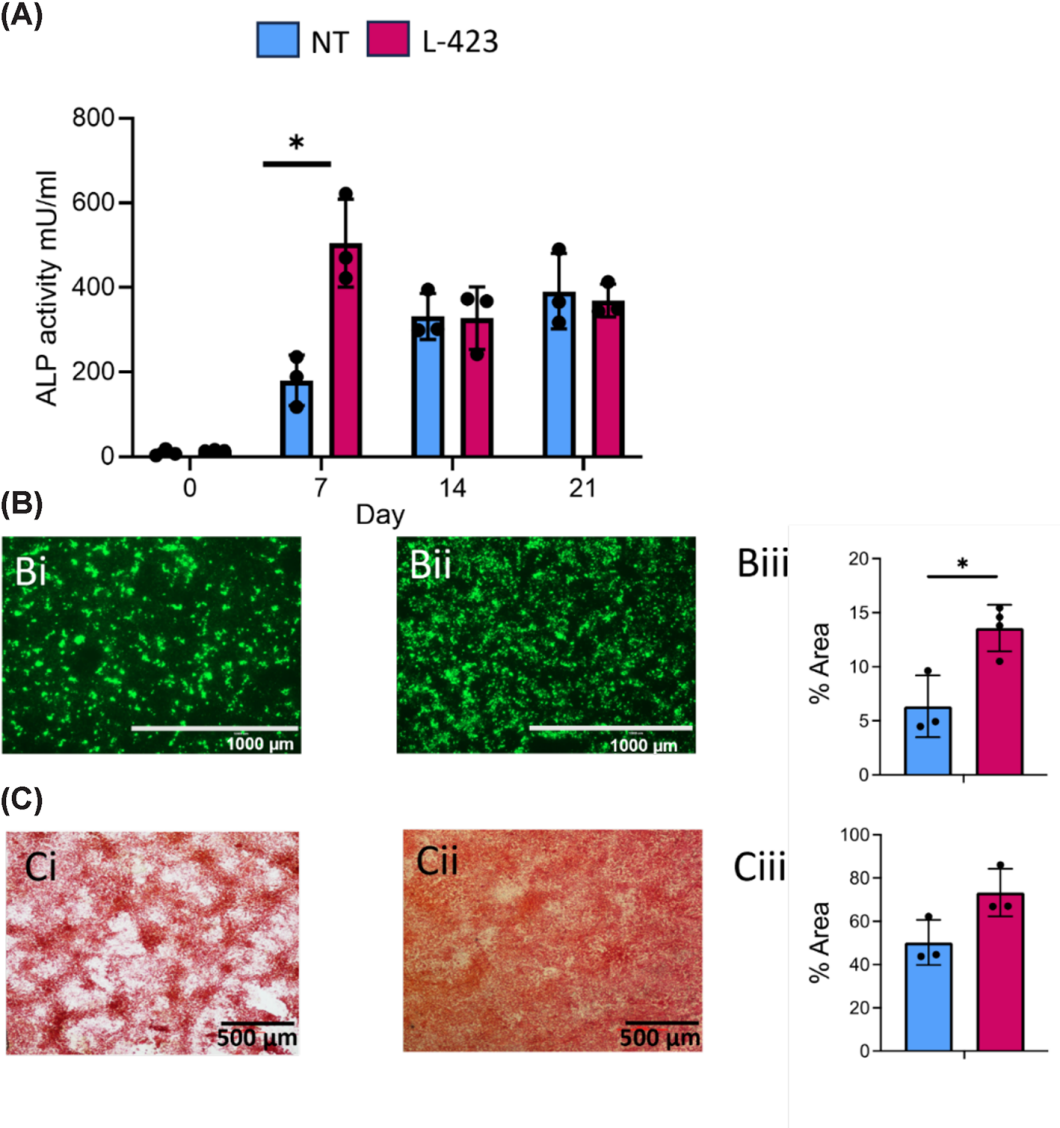
The effect of *LGASL1* knockdown on ALP activity, mineralisation, and matrix deposition (**A**) ALP activity measured in conditioned media taken at day 0 (basal culture) and after 7, 14, and 21 days in osteogenic media (NT = non-target control, L-423 = *LGALS1* knockdown cells). *indicates *P* <0.05 (mixed effects model with Bonferroni correction). (**B**) Staining of mineralised calcium phosphate crystals (green) after 21 days of osteogenic culture in (i) NT control, (ii) *LGALS1* knockdown cells. Scale bars = 1000 μm and (iii) quantification staining of mineralised calcium phosphate crystals showing the percentage area that is positively stained. *P <0.05 (unpaired student’s *t*-test). (**C**) Picrosirius staining for collagen after 21 days of osteogenic culture in (i) NT control, (ii) *LGALS1* knockout cells. Scale bars = 500 μm and (iii) quantification of staining showing the percentage area that is positively stained. Error bars represent standard deviation from three experimental replicates. No significant difference is observed (Mann–Whitney U test).

Knocking down *LGALS1* results in a significant increase in ALP activity under osteogenic culture at day 7 (Figure 8A). Furthermore, the amount of mineralisation after 21 days of osteogenic culture is significantly increased in cells that have reduced levels of *LGALS1* as measured using OsteoImage staining (for mineralised calcium phosphate crystals), demonstrating a significant two-fold increase (Figure 8Bi–iii). Picrosirius red staining for collagen shows no significant differences between control and *LGALS1* knockdown cells (Figure 8Ci–iii), despite a notable decrease in *COL1A1* expression following *LGALS1* knockdown and osteogenic culture.

## Discussion

LGALS1 has previously been studied in bone development and MSC differentiation. In this study the function of LGALS1 in mature osteoblasts was investigated. Saos2 cells were utilised due to their robust characteristics in culture, rapid proliferation, and ease of transfection. They have capacity to mineralize *in vitro* and have demonstrated a mature osteoblastic phenotype with the ability to transition towards an osteocyte-like phenotype when cultured under osteogenic conditions [38]. We acknowledge that Saos2 is an osteosarcoma cell line and may not fully recapitulate normal osteoblast-to-osteocyte differentiation. However, primary osteoblasts are not amenable to gene function studies due to their limited proliferation *in vitro* [39]. Immortalised primary cells may be advantageous in the future, but Saos2 cells have previously been used successfully to determine the function of genes in bone and mineralisation [40–44] and so were selected for use in the present study. In the first part of the present study, we further characterised Saos2 cells by performing a more comprehensive analysis of gene expression during basal and osteogenic culture. We demonstrated that under basal conditions, Saos2 cells express a combination of early osteoblast genes (*RUNX2* and *SP7*), mature osteoblast genes (*IBSP*, *ALPL*, *ENPP1*, *PHOSPHO1*, *BGLAP*, *COL1A1*, *SPARC*, and *SPP1*), late osteoblast/early osteocyte genes (*PHEX*, *MEPE*, and *DMP1*), and the late osteocyte marker *SOST*. We found that the expression of these genes changes during 21 days of culture in osteogenic media, with the cells exhibiting a gene expression profile that suggests that they have transitioned further towards an osteocyte-like phenotype. Previously, Prideaux et al. [38] demonstrated a small but significant reduction in *RUNX2* during osteogenic culture. However, in our study, *RUNX2* levels were fairly stable, and *SP7* (another early osteoblast gene) showed an increase in expression at days 14 and 21. Prideaux et al*.* [38], also showed a small but significant increase in *COL1A1* expression by day 14. Whereas in our data, we again demonstrated relatively stable *COL1A1* expression over time. These differences may reflect differences in the culture conditions; for example, we used a well-established combination of β-glycerophosphate, ascorbic acid, and dexamethasone [33], whereas the previous study used no dexamethasone and potassium dihydrogen phosphate [38]. Another study that used similar osteogenic culture conditions to us demonstrated that *RUNX2* was robustly expressed during osteogenic culture [34]. The other mature osteoblast genes we studied demonstrated either stable expression (*IBSP* and *SPARC*) or increased expression over the 21 days of culture (*BGLAP* and *SPP1*). Genes associated with the regulation of mineralisation showed peaks of increased expression at days 7 to 14 (*ALPL*, *ENPP1*, and *PHOSPHO1*). Importantly, we also confirmed previous work [34,38] that over 21 days of osteogenic culture, osteocyte markers (*PHEX*, *MEPE*, *DMP1*, and *SOST*) significantly increased in expression. Therefore, although Saos2 cells do not capture the full range of osteoblast phenotypes and are more restricted to the later stages of maturation [38], they provided a relevant cell type for this study. We next determined if *LGALS1* was required by Saos2 cells under basal (osteoblast) culture and for further osteogenesis. We demonstrated that *LGALS1* is expressed at high levels in Saos2 cells in both basal and osteogenic conditions. This was expected, as *LGALS1* has a relatively broad expression profile across multiple tissues and has been shown to be expressed in long-bone endocortical bone in young mice [45].

To determine the role of *LGALS1* in Saos2 cells, we modulated its expression and demonstrated that we could achieve significant overexpression and knockdown of *LGALS1* at the gene level under basal and osteogenic culture conditions. At the protein level, there was clear LGALS1 overexpression under basal conditions. However, under basal conditions secreted LGALS1 levels are low in the control cells, and therefore a robust knockdown of the protein was difficult to detect. However, under osteogenic conditions where protein levels are much higher, there is a significant knockdown of LGALS1 protein following the shRNA transduction. The ELISA protein data were not normalised to the number of cells present, and the increase in protein levels in osteogenic culture may simply reflect the increased number of cells present following the 21 days of culture, as mRNA levels did not increase under osteogenic conditions. A quantitative western blot would therefore be required to confirm the overexpression and knockdown at the protein level.

Following the successful modulation of LGALS1, we did not observe any significant effects on cell morphology (area and circularity) under basal culture conditions. Following osteogenic culture, the presence of the mineralised matrix and high cell density prevents cell morphology from being determined. However, modulation of LGALS1 caused a significant reduction in cell viability under basal culture. Both overexpression and knockdown significantly reduced cell viability, but there is a larger reduction when LGALS1 is knocked down (17% versus 47%). The reduction in viability could be due to a reduction in cell number from reduced cell proliferation, and this was not directly quantified in this study. *LGALS1* has been implicated in cell proliferation previously. In gastric epithelial cells, *LGALS1* knockdown reduced cell proliferation, and overexpression increased proliferation [46]. Similarly, the addition of LGALS1 to human BMSCs in culture was found to increase the proliferation [47]. Future work to directly measure the effects of LGALS1 modulation on osteoblast proliferation is therefore required. Unfortunately, it was not possible to measure cell viability under osteogenic culture due to the presence of the mineralised matrix, which prohibits the uptake of resazurin and prevents the isolation of single cells for cell counting. It may be possible to remove the calcium matrix deposits using EDTA [48] to allow the isolation of single cells to enable cell numbers and viability to be measured in the future.

We then determined whether LGALS1 modulation affected bone formation and mineralisation. Overexpressing LGALS1 caused a reduction in mineralisation in Saos2 cells, with alizarin red staining indicating reduced calcium deposition, which correlated with a trend for reduced ALP activity at day 14. The reduced mineralisation may be due to a decrease in cell number which could not be measured following osteogenic culture. Alizarin red staining binds to calcium rather than the mineralised calcium phosphate crystals and has a moderate sensitivity [49,50]. However, we were not able to measure mineralised calcium phosphate crystal deposition using the OsteoImage assay following LGALS1 overexpression as the vector used contained a GFP tag, which interfered with the assay readout. It is possible that the GFP tag used could affect LGALS1 function; however, the tagged protein was still secreted (as detected in our ELISA). Knocking down LGALS1 caused an increase in mineralisation characterised by an increase in mineralised calcium phosphate crystal deposition, which correlated with a significant increase in ALP activity at day 7. The increased mineralisation could be due to changes in cell numbers, which could not be measured following osteogenic culture; however, under basal conditions, LGALS1 knockdown decreased cell viability, whereas under osteogenic conditions it increased mineralisation. Furthermore, collagen deposition remained unaffected following LGALS1 modulation, suggesting that the observed changes in mineralisation were independent of collagen deposition. However, quantitative measurement of collagen deposition may have revealed smaller differences between the groups and other aspects of the matrix, such as proteoglycan content, were not measured in this study. Future work to measure protein levels of other matrix components would be beneficial. Together, these results suggest that LGALS1 is involved in negatively regulating mineralisation. These results are in contrast with previous work which was performed in BMSCs rather than osteoblasts, which demonstrated that LGALS1 overexpression resulted in increased calcium deposition and knockdown resulted in decreased calcium deposition [28]. There were some technical differences between these studies, with Chen et al. performing osteogenic culture for longer (28 days versus the 21 days we used) and using a higher concentration of β-glycerophosphate (10 versus 2 mM). However, we hypothesise that the different outcomes reflect the different development stages that the cells represent and that LGALS1 has different roles in early stem cell differentiation and in mature osteoblasts. This is not the first report of a regulator of osteogenesis having dual roles. For example, PPi acts to both inhibit and promote osteogenesis in a temporal pattern [22,51,52]. Likewise, magnesium can both promote and impair osteogenesis in a concentration-dependent manner [53]. And the transcription factor TRPS1 can both promote and repress mineralisation depending on the maturation stage of the cell [54].

To try to determine the mechanism of LGALS1 function in the Saos2 cells, we measured the expression of a panel of 14 osteogenic-associated genes. It has previously been shown that *LGALS1* binds to CD146 to activate Lrp5 expression and consequently the Wnt/β-catenin signalling pathway. This up-regulates *RUNX2* expression, which activates other genes, thus promoting differentiation of MSCs. Additionally, it was shown that up-regulating *RUNX2* expression results in strong calcium deposition and activation of *SPP1*, *IBSP*, and *BGLAP* [28]. However, in our study, overexpression of LGALS1 resulted in very few significant changes in gene expression under either basal or osteogenic culture conditions. Therefore, there were no clear alterations in either the differentiation state of the cells or in their expression of genes that are associated with mineralisation (*ALPL*, *ENPP1*, *PHOSPHO1*) that correlate with the decrease in mineralisation that we observed. Likewise, there were no significant changes in ALPL activity during the time course of the differentiation. In contrast, knocking down LGALS1 caused many changes in gene expression. Under basal conditions, many genes showed an increase in expression. But these genes represented a mix of early osteoblast (*SP7*), mature osteoblast (*SPP1*), and osteocyte markers (*PHEX*, *MEPE*, and *SOST*), and therefore there is not a clear shift in cell state following LGALS1 knockdown. Under osteogenic conditions there was a more mixed effect on gene expression, with some genes increasing in expression following LGALS1 knockdown (*RUNX2*, *ENPP1*, and *MEPE*) and other genes decreasing in expression (*SP7*, *COL1A1*, *DMP1*, and *SOST*). Therefore, there was again no obvious change in cell state following LGAS1 knockdown that would explain the increase in mineralisation that is observed. Similarly, there is no change in the expression of *ALPL* or *PHOSPHOP1*, which are regulators of matrix mineralisation. We did observe a significant increase in the expression of *ENPP1*. ENPP1 converts ATP to PPi [21], which, as described above, may both inhibit and promote osteogenesis. Therefore, there are no clear changes between the effect on gene expression and the increase in mineralisation that we observed following LGALS1 knockdown, and it is not possible to determine whether the changes in gene expression cause the changes in mineralisation. However, it is possible that a clearer link between gene expression and levels of mineralisation may have occurred at an earlier time point in the differentiation process, as we did observe a significant increase in ALP activity after 7 days of osteogenic differentiation following LGALS1 knockdown. We also only examined a relatively small number of genes, and future work to measure global transcriptional changes or protein-level changes may provide more insight into the role of LGALS1 in these cells.

Other limitations of the present study include the fact that we did not perform rescue experiments following LGALS1 knockdown or determine if multiple shRNA sequences to LGALS1 resulted in the same biological effects. It would have been advantageous to have performed this work to rule out the possibility that our observations are the result of off-target effects. Similarly, it should be noted that the modulations themselves have some effects, with both the non-target and empty vector controls reducing the expression of some osteogenic genes and reducing matrix mineralisation compared with the unaltered Saos2 cells (Supplementary Figures S4–6). This is not unexpected as the gene modulation process can affect cell properties due to a wide range of factors, including the integration site, the transfection reagent, and the selection antibiotic used [55]. This highlights the importance of comparing the gene modulations to relevant control cells [56]. However, to improve the validity of the results, it would be beneficial to include a second control shRNA with a different sequence and extra controls for the stable transfection (e.g. a no-plasmid control). We also did not examine the effect of LGALS1 modulation on mineralisation over time, and the use of different staining techniques to detect mineralisation following gene knockdown versus overexpression prevented direct comparisons from being made. In the future, direct measurements of mineralisation over time should also be correlated to cell numbers.

In conclusion, we have demonstrated that LGALS1 has a novel role in mature osteoblast-like cells. Future work to identify global gene expression profiles, collagen deposition, and mineralisation over time in response to LGALS1 modulation will help to define the biological processes and pathways that it regulates during the highly controlled and complex process of osteogenesis.

## Supplementary Material

Supplementary Figures S1-S7 and Tables S1-S2

## Data Availability

All data generated and analysed in this study are included in the published article and the supporting information.

## Abbreviations

ALPL: alkaline phosphatase
BGLAP: bone gamma carboxyglutamate protein
BMSCs: bone marrow stem/stromal cells
COL1A1: collagen type 1 aplha 1
DMP1: dentin matrix protein 1
IBSP: bone sialoprotein
ENPP1: ectonucleotide pyrophosphatase/phosphodiesterase-1
M-CSF: macrophage colony stimulating factor
MEPE: matrix extracellular phosphoglycoprotein
MSCs: mesenchymal stromal cells
NHS: normal horse serum
PBS: phosphate buffered saline
PHEX: phosphatase-regulating neutral endopeptidase
PHOSPHO1: bone-specific phosphatase (phosphatase, orphan 1)
Pi: inorganic phosphate
PPi: inorganic pyrophosphate
RANKL: receptor activator of nuclear factor kappa-B ligand
RUNX2: runt-related transcription factor 2
SOST: sclerostin
SP7: SP7 transcription factor (osterix)
SPP1: secreted phosphoprotein 1 (osteopontin)
