# Supplementary material for: Modulating Galectin-1 in human osteoblast-like cells alters mineralisation and influences the expression of genes associated with osteoblasts and osteocytes
Source: Biosci Rep. 2026 Feb 23;46(3):BSR20253890. doi: 10.1042/BSR20253890 (PMC13071376; doi:10.1042/BSR20253890)
Supplement: Supplementary Figures S1-S7 and Tables S1-S2 [file BSR-2025-3890_supp.pdf]

**Supporting information**

**Table S1:** Comprehensive ranking of candidate housekeeping genes for qPCR assays using unaltered Saos2 cells under basal and osteogenic conditions; ACTB is the most stable

| Gene | Comprehensive ranking | | Delta Ct | | geNorm | | Normfinder | | Bestkeeper | |
|---|---|---|---|---|---|---|---|---|---|---|
| | Value | Rank | SD Average | Rank | M value | Rank | Stability | Rank | SD | Rank |
| ACTB | 1.00 | 1 | 0.680 | 1 | 0.350 | 1 | 0.045 | 1 | 0.600 | 1 |
| B2M | 4.00 | 4 | 1.050 | 4 | 0.847 | 3 | 0.943 | 4 | 0.970 | 4 |
| GAPDH | 1.86 | 2 | 0.730 | 2 | 0.350 | 1 | 0.315 | 2 | 0.680 | 3 |
| RPS18 | 2.71 | 3 | 0.940 | 3 | 0.643 | 2 | 0.775 | 3 | 0.670 | 2 |

**Table S2.** List of primer sequences used in qPCR.

| **Gene** | **Protein Name** | **Gene ID** | **Forward Primer** | **Reverse Primer** |
|---|---|---|---|---|
| *ACTB* | Actin Beta | 60 | GCCTCGCCTTTGCCGAT | CGCGGCGATATCATCATCC |
| *ALPL* | Alkaline Phosphatase | 249 | CACGGAACTCCTGACCCTTG | TCCTGTTCAGCTCGTACTGC |
| *BGLAP* | Bone gamma-carboxyglutamate protein or Osteocalcin | 632 | CACTCCTCGCCCTATTGGC | CCCTCCTGCTTGGACACAAAG |
| *COL1A1* | Collagen Type 1 Alpha 1 Chain | 1277 | GCCCTGCTGGTGCTCG | GGACCTTCAGAGCCTCGGG |
| *DMP1* | Dentin Matrix Acidic Phosphoprotein 1 | 1758 | AGTGGCTTCATTGGGCATAGA | TCCCCAAAGGAACATGAGCAG |
| *ENPP1* | Ectonucleotide pyrophosphatase/ phosphodiesterase 1 | 5167 | CCCTCAGTGGCAACTTGCAT | GCCAACAAAGAGGGCTTGC |
| *IBSP* | Integrin Binding Sialoprotein | 3381 | AGGACTGCCAGAGGAAGCAA | GAGAAAGCACAGGCCATTCC |
| *LGALS1* | Galectin 1 | 3956 | TGACGCTAAGAGCTTCGTGC | CGTTGAAGCGAGGGTTGAAG |
| *MEPE* | Matrix Extracellular Phosphoglycoprotein | 56955 | TCCTTTTCAGTGTGACCTGGG | TCCACACAGCTTTGCTTAGT |
| *PHEX* | Phosphate Regulating endopeptidase X-Linked | 5251 | ACCTTAGCAGGCGCTTTCAG | TTTGTCCCATTGAGGCAGCA |
| *PHOSPHO1* | Phosphoethanolamine/ phosphocholine phosphatase 1 | 162466 | GTAAGCACCCCTTGCTCCAT | GTCACACGTTCGTGGGGG |
| *RUNX2* | RUNX Family Transcription Factor 2 | 860 | ACCGAGACCAACAGAGTCATTTA | GTCACTGTGCTGAAGAGGCT |

| | | | | |
|---|---|---|---|---|
| *SOST* | Sclerostin | 50964 | CACCACCCCTTTGAGACCAA | GTCACGTAGCGGGTGAAGT |
| *SPARC* | Secreted Protein Acidic And Cysteine Rich | 6678 | CAAGAAGCCCTGCCTGATGA | TCTTCGGTTTCCTCTGCACC |
| *SPP1* | Secreted phosphoprotein 1/ osteopontin | 6696 | AGCAGAATCTCCTAGCCCCA | TGGTCATGGCTTTCGTTGGA |
| *SP7* | Osterix | 121340 | GCCATTCTGGGCTTGGGTAT | TGCAGGTATCAGGCACAAGG |

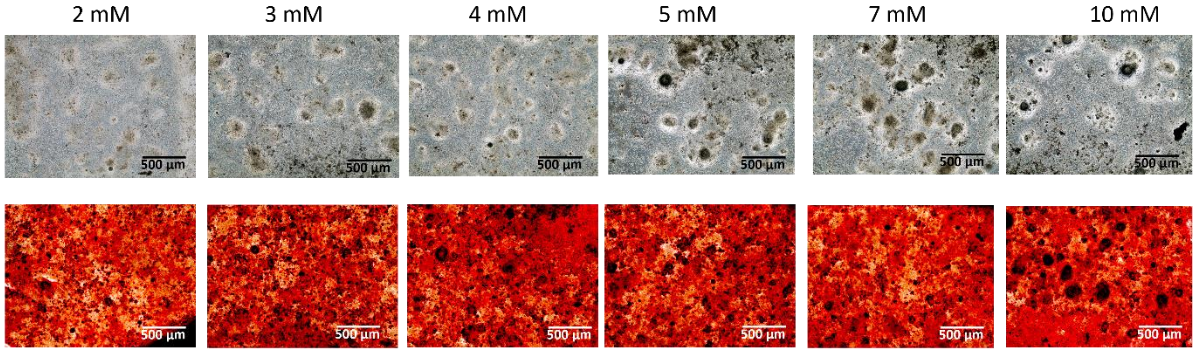


**Supplementary Figure 1.** BGP optimisation on unaltered Saos2 cells. Top row = pre-staining, bottom row = post-staining with Alizarin red. Scale bars = 500 µm. Images representative of three experimental replicates.

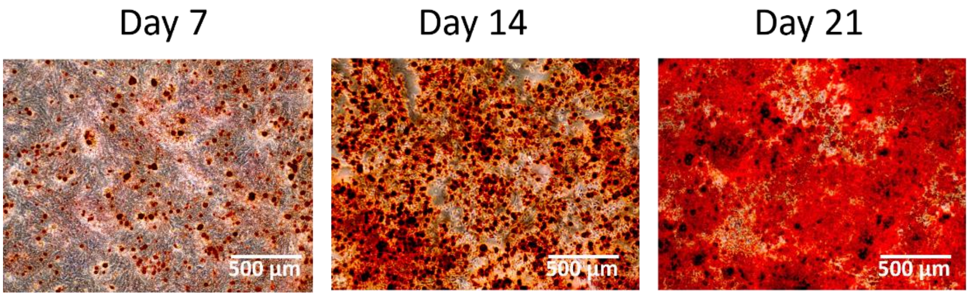


**Supplementary Figure 2. Alizarin red staininING on unaltered Saos2 cells at different time points during osteogenic culture.** Scale bars = 500 µm.

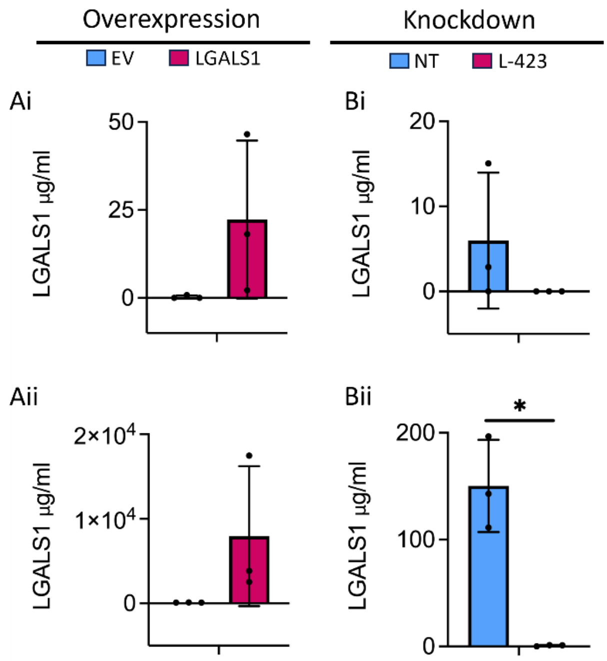


**Supplementary Figure 3. LGALS1 protein modulation in Saos2 cells.** A) Overexpression under basal (i) and osteogenic (ii) conditions. B) Knockdown under basal (i) and osteogenic (ii) conditions. EV= empty vector, LGALS1 = LGALS1 overexpressing cells, NT = non-target control, L-423 = LGALS1 knockdown. The error bars represent the standard deviation from 3-5 experimental replicates**.** *indicates $p<0.05$. Data in A and B were analysed with a Mann Whitney U test. Data in C and D were analysed with a Student's unpaired T-test with Welch's correction.

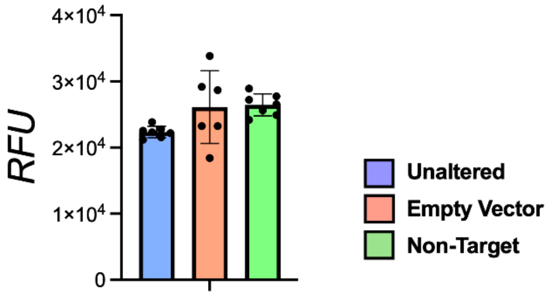


**Supplementary Figure 4.** Cell viability of unaltered Saos2, overexpression modulation control (Empty Vector) and knockdown modulation control (Non-Target) cells cultured under basal conditions. Cell viability is shown as relative fluorescent units (RFU). Unaltered cells (blue), empty vector control (orange), non-target control (green). Error bars represent the standard deviation from 7 technical replicates. (Brown-Forsythe ANOVA).

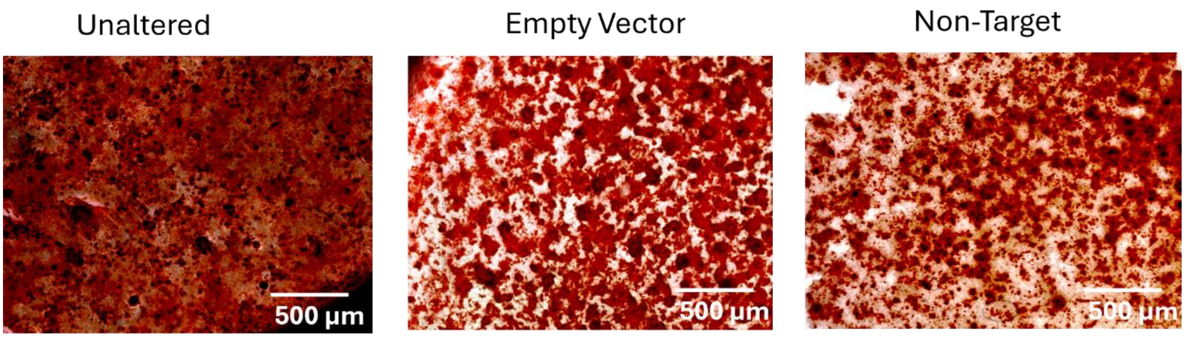


**Supplementary Figure 5.** Matrix mineralisation by unaltered Saos2, overexpression modulation control (Empty Vector) and knockdown modulation control (Non-Target) cells cultured under osteogenic conditions for 21 days and stained with Alizarin red. Scale bars = 500 µm. Images representative of three experimental replicates.

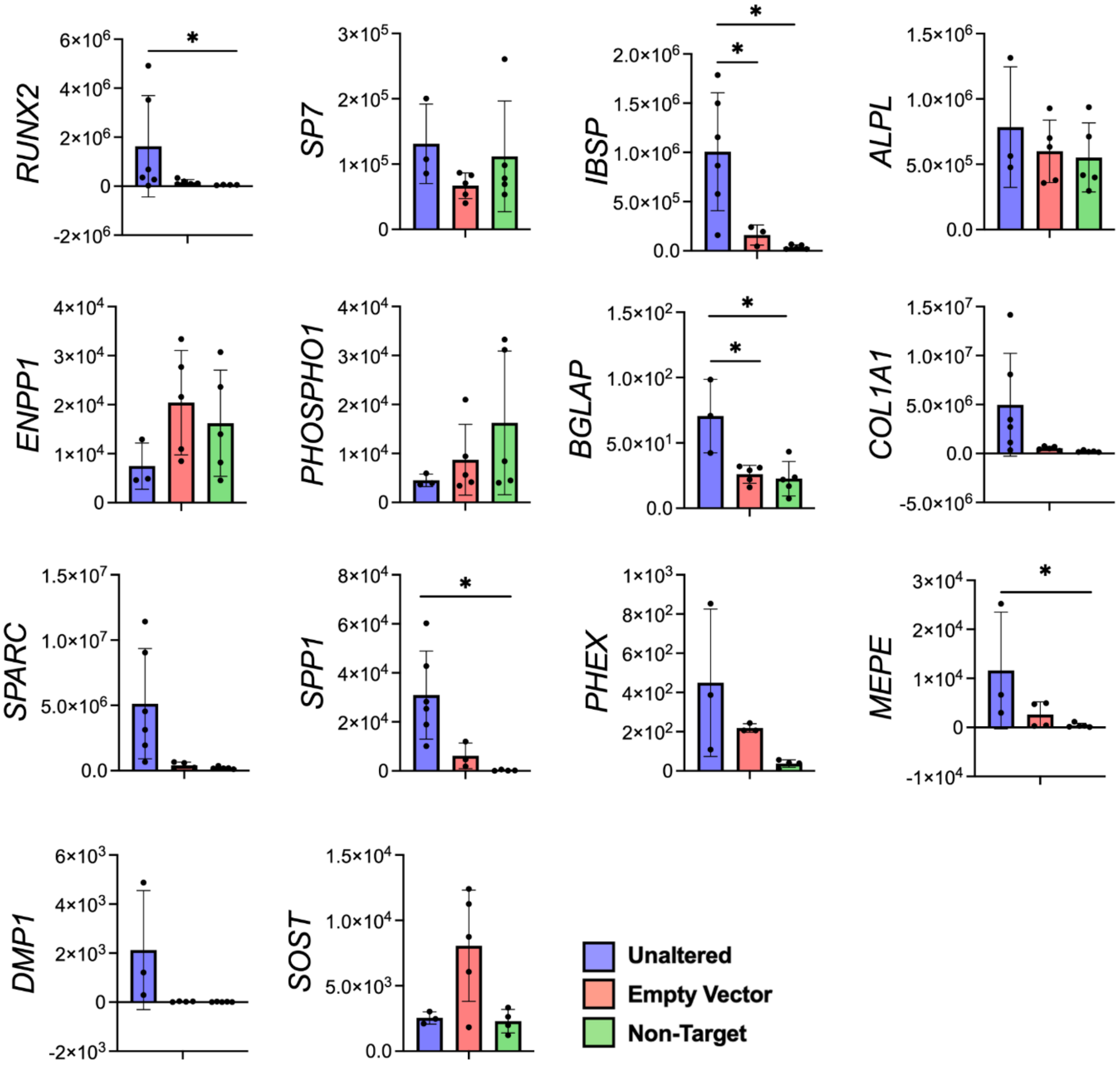

**Supplementary Figure 6.** Expression of osteogenic genes in control cell types cultured under basal conditions. Unaltered cells (blue), empty vector control (orange), non-target control (green). Expression is shown relative to the *ACTB* housekeeping gene. Error bars represent the standard deviation from three to five experimental replicates. * indicates p<0.05 compared to the unaltered cells (One way ANOVA with Tukey's post hoc test, or where expression was not normally distributed, a Kruskal-Wallis with Dunn's post-hoc test).

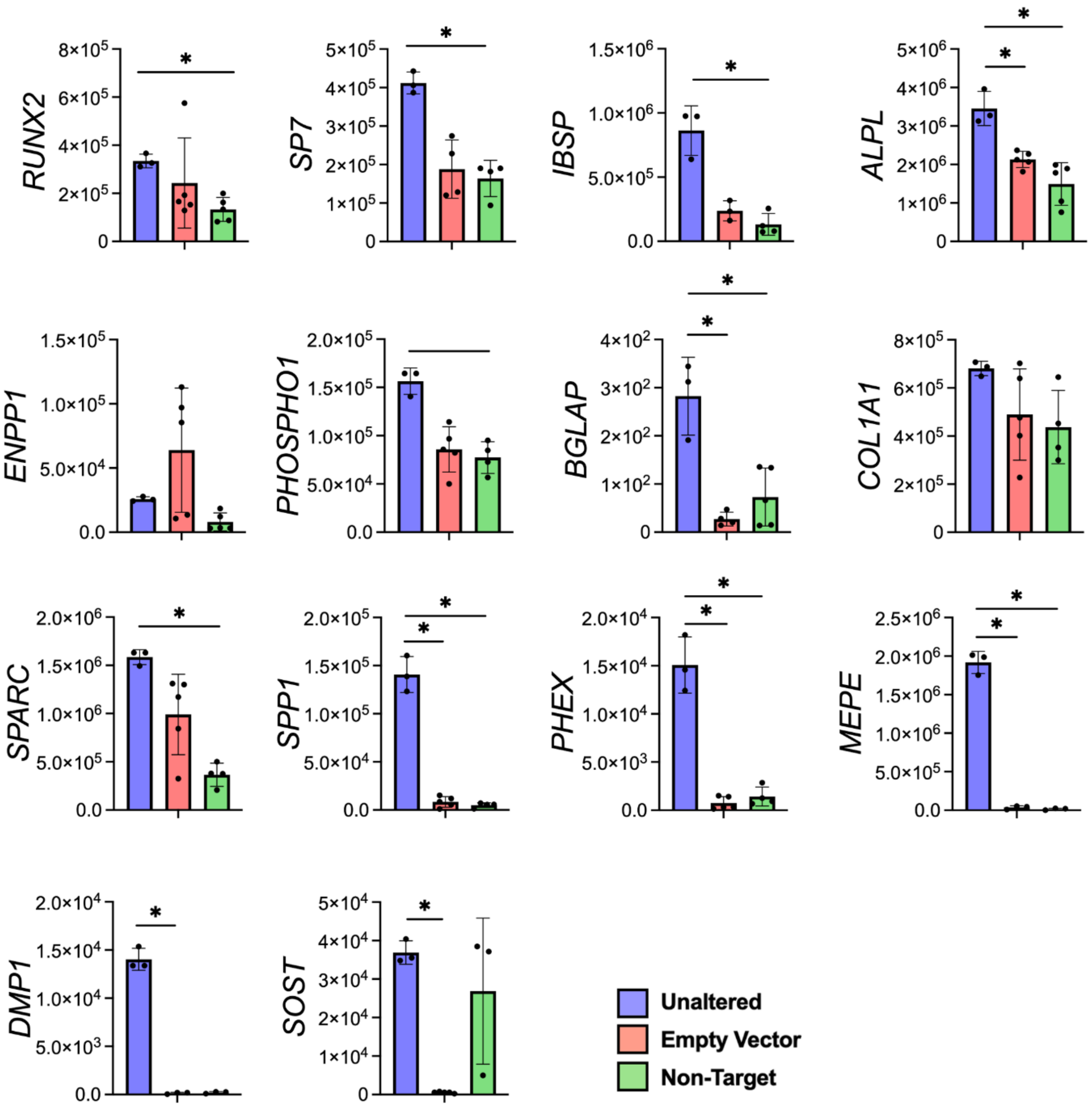


**Supplementary Figure 7.** Expression of osteogenic genes in control cell types cultured under osteogenic conditions. Unaltered cells (blue), empty vector control (orange), non-target control (green). Expression is shown relative to the *ACTB* housekeeping gene. Error bars represent the standard deviation from three to five experimental replicates. * indicates p<0.05 compared to the unaltered cells (One way ANOVA with Tukey's post hoc test, or where expression was not normally distributed, a Kruskal-Wallis with Dunn's post-hoc test).
